# Correlating electroconvulsive therapy response to electroencephalographic markers: Study protocol

**DOI:** 10.3389/fpsyt.2022.996733

**Published:** 2022-11-03

**Authors:** Subha Subramanian, Alyssa K. Labonte, Thomas Nguyen, Anhthi H. Luong, Orlandrea Hyche, S. Kendall Smith, R. Edward Hogan, Nuri B. Farber, Ben Julian A. Palanca, MohammadMehdi Kafashan, CET-REM Study Group, Gaurang Gupte

**Affiliations:** ^1^Department of Psychiatry, Washington University School of Medicine in St. Louis, St. Louis, MO, United States; ^2^Department of Neurology, Berenson-Allen Center for Noninvasive Brain Stimulation, Beth Israel Deaconess Medical Center, Boston, MA, United States; ^3^Department of Psychiatry, Beth Israel Deaconess Medical Center and Harvard Medical School, Boston, MA, United States; ^4^Department of Anesthesiology, Washington University School of Medicine in St. Louis, St. Louis, MO, United States; ^5^Neuroscience Graduate Program, Washington University School of Medicine in St. Louis, St. Louis, MO, United States; ^6^Department of Health Policy and Management, Columbia University, New York, NY, United States; ^7^Center on Biological Rhythms and Sleep, Washington University School of Medicine in St. Louis, MO, United States; ^8^Department of Neurology, Washington University School of Medicine in St. Louis, St. Louis, MO, United States; ^9^Division of Biology and Biomedical Sciences, Washington University School of Medicine in St. Louis, St. Louis, MO, United States; ^10^Department of Biomedical Engineering, Washington University in St. Louis, St. Louis, MO, United States; ^11^Neuroimaging Labs Research Center, Washington University School of Medicine in St. Louis, St. Louis, MO, United States

**Keywords:** electroencephalography (EEG), depression, electroconvulsive therapy (ECT), sleep, slow wave (NREM) sleep, sleep spindle, seizure

## Abstract

**Introduction:**

Electroconvulsive therapy (ECT) is an effective intervention for patients with major depressive disorder (MDD). Despite longstanding use, the underlying mechanisms of ECT are unknown, and there are no objective prognostic biomarkers that are routinely used for ECT response. Two electroencephalographic (EEG) markers, sleep slow waves and sleep spindles, could address these needs. Both sleep microstructure EEG markers are associated with synaptic plasticity, implicated in memory consolidation, and have reduced expression in depressed individuals. We hypothesize that ECT alleviates depression through enhanced expression of sleep slow waves and sleep spindles, thereby facilitating synaptic reconfiguration in pathologic neural circuits.

**Methods:**

Correlating ECT Response to EEG Markers (CET-REM) is a single-center, prospective, observational investigation. Wireless wearable headbands with dry EEG electrodes will be utilized for at-home unattended sleep studies to allow calculation of quantitative measures of sleep slow waves (EEG SWA, 0.5–4 Hz power) and sleep spindles (density in number/minute). High-density EEG data will be acquired during ECT to quantify seizure markers.

**Discussion:**

This innovative study focuses on the longitudinal relationships of sleep microstructure and ECT seizure markers over the treatment course. We anticipate that the results from this study will improve our understanding of ECT.

## Highlights

- Electroconvulsive therapy (ECT) is a highly effective treatment for depression.
- It is unclear how ECT perturbs brain circuitry.
- There are currently no objective markers to prognosticate the likelihood of ECT response.
- Assessing longitudinal changes in sleep microstructure–sleep slow waves and sleep spindles–may aid in elucidating neuroplasticity during the ECT course.
- We hypothesize that ECT alters expression of sleep spindles and sleep slow waves to facilitate synaptic neural reconfiguration.
- We will evaluate the following markers acquired throughout the ECT course: (1) sleep EEG markers from at-home sleep studies and (2) high-density EEG acquired during generalized seizures that can be localized to neural circuits.

## Introduction

### Electroconvulsive therapy: An effective treatment for depression but with unclear underlying mechanisms

Electroconvulsive therapy (ECT) is an effective treatment for major depressive disorder (MDD), a common illness ranking as the second leading cause of disability worldwide (1). During ECT, a brief electrical stimulus is delivered through the scalp to elicit a generalized seizure under general anesthesia. Two to three ECT sessions are administered per week during the index treatment course. Compared to oral antidepressants, ECT ameliorates depressive symptoms rapidly; some individuals experience improvement shortly after the third treatment (2). The response rate to ECT for a depressive episode is up to 80% (2–4), with remission rates ranging from 50 to 63% (5, 6).

Electroconvulsive therapy was used for the first time on a human patient in 1938 by Cerletti and Bini (7). Although ECT has been used for over 80 years, many unknowns remain. It is thought that ECT induces neuroplasticity and alters neural connectivity within functional networks; however, these networks have yet to be elucidated (8, 9). Moreover, it remains unclear why certain patients do not respond to therapy. Prognostic physiologic biomarkers are not routinely used to predict and monitor ECT response. Identifying such biomarkers may aid clinicians in referring the right candidates for ECT or adapting ECT parameters (i.e., charge and electrode placement) to improve treatment response.

### Probing relationships between sleep abnormalities and major depressive disorder

Sleep disturbance is prevalent in those with depression (10). Greek physicians in the 1600s described the co-occurrence of insomnia and depression in the *Anatomy of Melancholia* (11), as did the founder of modern psychiatry, Kraepelin, in the early 1900s (12). Between 60 and 90% of patients with MDD are afflicted by symptoms of delayed sleep onset, shorter sleep duration, and nonrestorative sleep (13, 14). Those with severe MDD sub-types and increased suicidal ideation are more likely to have poor sleep patterns (15). Similarly, individuals with sleep disturbances are at a higher risk of developing depressive symptoms (16).

Given this interaction between abnormal sleep and depression, previous investigations have relied on polysomnography (PSG) to study sleep patterns. PSG utilizes electroencephalography (EEG) and other physiologic signals to segment sleep into periods of rapid eye movement (REM) and stages of non-rapid eye movement (NREM) sleep (17). NREM stage 2 (N2) constitutes most of total sleep, while NREM stage 3 (N3/slow wave sleep) is associated with restorative physiologic benefits across multiple organ systems (18–20). Patterns of sleep disturbance associated with MDD, such as increased REM duration and shortened REM latency (21–23), have been considered as potential targets for monitoring antidepressant response (24).

**FIGURE 1:**
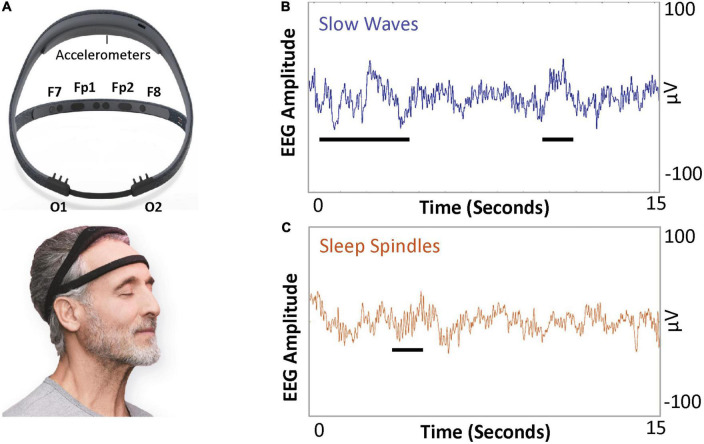
Dreem device and example illustration of sleep slow wave activity (SWA) and spindles. **(A)** The Dreem device is a consumer-grade, wireless EEG device. The device has three frontal EEG channels, at F7, F8, and Fpz, and two occipital channels at O1 and O2. Both frontal and occipital channels are sufficient to allow quantification of SWA (average power in 0.5–4 Hz frequency band per minute) and sleep spindle density (number of spindles a minute). **(B)** Slow waves (0.5–4 Hz frequency) and **(C)** sleep spindles (11–16 Hz frequency band).

Polysomnography usage in longitudinal studies has the following limitations: requirement of wire electrodes and sensors for monitoring; perturbations in natural sleep that may occur in a foreign environment, such as a sleep clinic; and limited scalability for large clinical study designs. Newer technology can circumvent such logistical impediments of PSG to allow longitudinal assessment of sleep structure over the course of ECT. The Dreem device (Figure 1A, Dreem, Paris, France) is a consumer-grade, wireless, dry-electrode headband that records EEG without the need for a continuous internet or Bluetooth connection (25). This device has previously been validated against polysomnography for monitoring sleep-related physiological signals and accurate sleep staging across broad age demographic (26). As the device has been suitable for longitudinal use in the perioperative setting (27), the Dreem device may be a feasible option for obtaining continuous sleep EEG data over an ECT course.

### Sleep electroencephalographic markers of depression, antidepressant response, and synaptic plasticity

Two sleep EEG markers of synaptic plasticity, sleep slow waves, and sleep spindles, are pertinent to depression and antidepressant response.

Sleep slow waves (Figure 1B) are high-amplitude low-frequency oscillations (<4 Hz) with predominance during N3 sleep. Sleep slow wave activity (SWA) is quantified as the EEG power per minute in the 0.5–4 Hz frequency band. SWA is a physiologic marker of the homeostatic sleep pressure, as SWA increases during the day and decreases during NREM sleep (28–30). SWA during N3 sleep is reduced in the first cycle of sleep in depressed individuals versus healthy controls (31–33). Additionally, sleep slow waves may have a lower amplitude in patients with depression compared to healthy controls (34). Dissipation of SWA, measured as the decline of SWA throughout sleep, may also be an important marker. The delta sleep ratio (DSR; quotient of SWA during first N3 cycle SWA to SWA during the second N3 cycle) is low in patients with depression (35, 36).

Antidepressant therapies such as ketamine and sleep deprivation enhance subsequent sleep SWA in depressed individuals. Duncan et al. demonstrated that SWA in the first N3 cycle increased after subanesthetic ketamine infusion and was correlated with an improvement in depressive symptoms (37, 38). Sleep deprivation, which can confer short-lived antidepressant effects in a subset of MDD patients, leads to a subsequent rebound of sleep SWA (39), with a high DSR predicting antidepressant response (36). Altogether, these findings suggest that sleep SWA measures could be useful in monitoring antidepressant response.

Slow wave activity has also been linked to neural plasticity (40, 41), a key neuronal adaptation mechanism putatively impaired in depression (42–44). In rodents, expression of sleep SWA correlates with extent of environmental exploration (45), with sleep SWA augmented by exogenous brain-derived neurotrophic factor (BDNF) (44). BDNF is involved in the control of synapse formation and regulation (46–48), and its dysfunction leads to synaptic plasticity malfunction (49, 50). Neural plasticity involves changes in synaptic connections or remodeling of dendrites. Loss of dendritic branching and spine complexity have been shown in human postmortem hippocampal tissue in depressed individuals (51). These impairments in neuroplasticity have been rescued by rapid antidepressants *via* the neurotrophic cascade (52). Sleep may be an important time for this synaptic remodeling of connections built during the wakeful conscious experience (53) with sleep SWA as a marker of net cortical synaptic strength. Facilitation of synaptic plasticity in memory reconsolidation involves temporal coordination of sleep slow waves and sleep spindles (54–57).

Sleep spindles (Figure 1C) are 11–16 Hz crescendo-decrescendo oscillatory signatures that define entry into N2 sleep. Sleep spindles originate from circuits that include the thalamic reticular nucleus and the neocortex (58). Spindles are hypothesized to stabilize sleep and are associated with consolidation of memory during sleep (59–62). It is hypothesized that depression is linked to poor memory for positive stimuli, leading to a void of positive memories and anhedonia (63). Moreover, individuals with MDD have impairments in memory consolidation (64) and a reduction of spindle density, defined as the number of detected spindles per minute (65, 66). Decreased sleep spindle density is also observed in response to stress (67). While decreased spindle density is associated with depression, it remains unclear if and how antidepressants regulate sleep spindles.

Given the interrelatedness among SWA, sleep spindles, depression, and neuroplasticity (68), it is plausible that SWA and sleep spindles may serve as biomarkers of synaptic reconfiguration induced during responses to ECT and other antidepressants (29).

### Electroconvulsive therapy alters sleep macrostructure, but changes in sleep microstructure remain uncharacterized

A few studies have examined longitudinal changes in sleep architecture in response to ECT. These have focused on measures of sleep macrostructure, including durations or latencies of REM or N3 sleep. Most studies have examined ECT-induced changes in sleep EEG by comparing sleep macrostructure before and after an entire treatment course, with discordant results. A full course of ECT is associated with increases in daytime activity, total sleep time, and sleep efficiency (69). Right unilateral ECT increased the proportion of REM and N3 sleep in a group of 15 adult patients who showed remission from depression (70). However, another set of studies reported no significant association between REM sleep parameters when comparing ECT responders and non-responders (71–73). One concern is that sleep macrostructure markers are impacted by variability in daytime somnolence and artifacts that impair sleep staging. In contrast, sleep SWA and sleep spindle density are quantitative EEG metrics that may be more robust to such sources of variance. How sleep spindle density or sleep SWA evolves over an ECT course remains unknown.

### Electroconvulsive therapy induces seizure markers linked to sleep circuitry

While induction of a seizure is required for an antidepressant response, criteria for an efficacious seizure remain unknown. Initially, longer seizure duration was associated with increased effectiveness; however, this relationship has not held (74, 75). Thus, there is growing interest in identifying objective seizure quality markers for prognosticating clinical outcomes. Several EEG characteristics have been employed to predict response to ECT, including postictal suppression (76, 77) and ictal amplitude measures (78). Yet, such EEG measurements have not been integrated into clinical practice as biomarkers of ECT efficacy (79).

**FIGURE 2:**
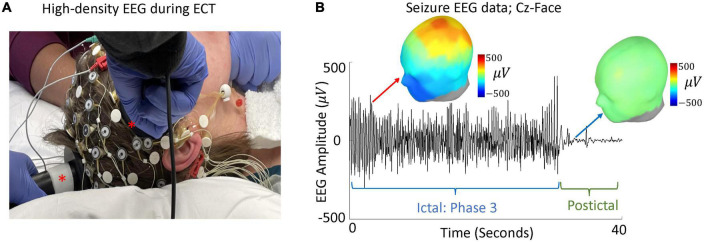
High-density EEG during ECT and evaluation of CPCs. **(A)** A modified high-density 64-electrode EEG net with right unilateral ECT placement (ECT paddles marked with asterisks). **(B)** Data from a single seizure, during phase 3 and 10 s after seizure termination, is shown. EEG amplitude between Cz and suborbital facial electrodes (Cz-Face) is used to detect large amplitude CPCs. Green line shows seizure termination and onset of postictal generalized EEG suppression (PGES) (103, 104). Topographic maps represent spatial topology of EEG voltage during phase 3 (red arrow) and PGES (blue arrow).

Through evaluation of high-density EEG acquired during right unilateral ECT (Figure 2A) (80), our group detected an ECT-induced ictal wave form during associated generalized seizures. These central-positive complexes (CPC) (81) are large in amplitude (hundreds of microvolts) with a descending gradient of positive electrical charge from the vertex of the scalp (Figure 2B). During ECT-induced seizures, CPCs correlate with bilateral clonic activity, qualitatively decreasing in frequency over time (81). Peak amplitude of CPCs are localized to the cingulate cortex and medial dorsal thalamus (82)—structures involved in the formation of sleep slow waves and sleep spindles (64, 83–85). Thus, CPCs could be involved in the alteration of SWA and sleep spindles, but the relationships between CPCs, sleep slow waves, and sleep spindles throughout treatment course requires further elucidation.

### Objectives and specific aims

The primary objective of our study is to longitudinally track changes in sleep microstructure throughout the ECT course of a convenience sample of patients, who are clinically ascertained and treated for depressive episodes on a busy hospital-based service. Wearable dry-electrode EEG devices will be utilized to monitor overnight sleep at home. High-density EEG during the ECT procedure will allow characterization of seizure markers. Specific aims, hypotheses, and analytical approaches are listed below.

#### Specific aim 1

Assess changes in N2 and N3 sleep SWA over the course of serial ECT sessions. We hypothesize SWA will increase over the course of ECT. Separate linear mixed effects models will be constructed to assess SWA changes in responder and non-responder groups.

#### Specific aim 2

Evaluate changes in sleep spindle density during N2 and N3 sleep over the course of ECT. We predict that there will be an increase in sleep spindle density throughout ECT. Separate linear mixed effects models will be constructed to assess changes in sleep spindle density in responder and non-responder groups.

#### Exploratory aim

Characterize the relationship between changes in CPCs and sleep SWA and spindle density. We hypothesize that the cumulative duration of CPCs will positively correlate with SWA and sleep spindle density. Separate linear mixed effects models will be constructed to test this hypothesis in responder and non-responder groups.

## Methods and analysis

### Study design

**FIGURE 3:**
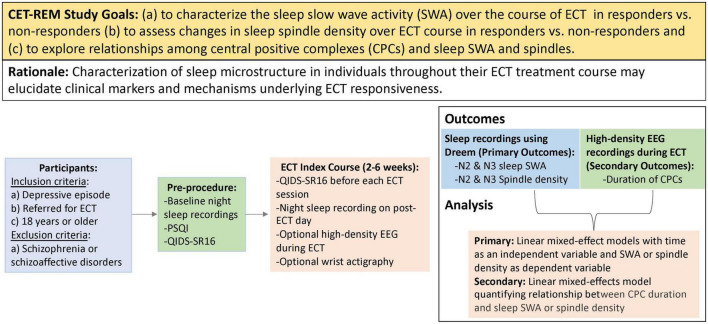
PICOT (participants, intervention, control, outcomes, timing) summary of study design. This study will recruit adults requiring ECT treatment for a depressive episode. Participants will wear a Dreem headband at night to obtain sleep EEG recordings throughout the course of ECT. High-density EEG recordings will also be obtained during ECT sessions to assess central positive complexes (CPCs). The primary outcomes of CET-REM include characterization of slow wave activity and sleep spindle density throughout ECT course.

Correlating Electroconvulsive Therapy Response to Electroencephalographic Markers (CET-REM) is a single center, prospective, observational study (ClinicalTrials.gov: NCT04451135) in a clinically ascertained and treated sample. See Figure 3 for study design. Inclusion criteria include planned ECT, age 18 and above, presence of a depressive episode (either unipolar or bipolar depressive episode). Exclusion criteria include schizophrenia and schizoaffective disorder.

Each enrolled participant will undergo an index course of ECT, following standard clinical care. Self-reported depressive scores will be assessed prior to each ECT session to determine response, and the total number of treatments will be determined by the psychiatrist performing ECT, as part of routine clinical practice. Response to the ECT will be defined as a 50% reduction of baseline QIDS score. Sleep structure on the nights of ECT sessions would be confounded by the effects of the anesthetic used in the procedure. Thus, ambulatory overnight sleep EEG data will be acquired *via* a Dreem device on post-ECT days. For exploratory analyses, optional high-density EEG data will be recorded during ECT-induced seizures. Our study design will allow us to longitudinally collect a large data set of EEG markers, including sleep slow waves, sleep spindles, and CPCs.

### Participant recruitment

Twenty-five patients with depression (either unipolar or bipolar depressive episode) will be recruited from the Barnes-Jewish Hospital inpatient floors or the ECT-outpatient referral service. Consulting ECT psychiatrists will interview potential participants to determine diagnosis, eligibility, and interest in the study. As per routine practice, Diagnostic and Statistical Manual of Mental Disorders (DSM-5) criteria will be utilized by the ECT clinician in determining the diagnosis. Suitable study candidates will be referred by ECT psychiatrists to study coordinators. Informed consent will be obtained. Once enrolled, study participants will receive a paper consent form and study material directing participants to the ClinicalTrials.gov record.^1^

### Clinical assessments

Two clinical assessments will be done throughout this study, the QIDS-SR16 (Quick Inventory of Depressive Symptomatology-16 Self Report), and the PSQI (Pittsburgh Sleep Quality Index). The QIDS-SR16 measures the participant’s depressive symptoms over the past seven days (86). At our institution, patients receiving ECT typically complete QIDS-SR16 throughout their ECT course. While other scales for measuring depression severity exist, such as Hamilton Depression Rating Scale (HAMD) and Montgomery–Asberg Depression Rating Scale (MADRS), the usage of QIDS-SR16 in the ECT suite has been historically chosen because it is a patient-reported assessment that accommodates for the high volume of participants in the suite limited clinician resources. The questionnaire includes 16 questions that survey nine symptom domains: sad mood, self-criticism, poor concentration, suicidal ideation, anhedonia, energy, sleep disturbance, appetite, and psychomotor agitation. Paper versions of the QIDS-SR16 will be completed by participants prior to each ECT session. The PSQI is a scoring system to assess sleep quality and habits during the past month (87). The assessment measures seven components of sleep: subjective sleep quality, sleep latency, sleep duration, sleep efficiency, sleep disturbances, sleeping medications, and daytime dysfunction. PSQI will be obtained *via* an electronic form administered prior to the first ECT session. Suicidality will be assessed by psychiatry physicians in the ECT suite, as per standard of care.

### Electroconvulsive therapy

Electroconvulsive therapy will be delivered using standard-of-care clinical protocols. Three stimulation electrode placements will be utilized: right unilateral (electrodes placed on the right temple and vertex of the head), bilateral (electrodes placed on the right and left temple), and bifrontal (both electrodes placed on the forehead, above the outer canthus of the eye).

Prior to ECT stimulation, general anesthesia will be induced. Commonly used regimens include etomidate 0.2 mg/kg, methohexital 1 mg/kg, or ketamine 1.5 mg/kg. The anesthetic regimen will be determined at the anesthesiologist’s discretion. Standard American Society of Anesthesiologists safety monitoring will occur throughout treatment.

The ECT stimulation will be delivered *via* routine clinical practice, using the Thymatron System IV. On the initial treatment (dose titration) session, ECT-naïve individuals typically receive right unilateral ECT titration at 5% charge, 0.3 ms pulse-width, 40 Hz frequency. While a seizure duration of 20–25 s has been accepted to be adequate by some, there are no firm data that support this position. Instead, seizure duration appears to not determine adequacy of response (88–93). Therefore, at our institution seizure duration is not used to determine adequacy of the treatment. Instead, a seizure duration of 10 s is defined as a reasonable minimal limit for determining the presence of an induced seizure. If an appropriate seizure is not obtained after this initial stimulation, then ECT charge will be increased in a protocolized fashion until a seizure of at least 10 s is obtained. Subsequent right unilateral treatments are then administered at a charge sixfold above seizure threshold.

If, based upon the clinical judgment of the ECT clinician, right unilateral ECT at sixfold seizure threshold does not improve depressive symptoms, participants may then transition to 100% right unilateral charge and if need be bilateral ECT at two-times seizure threshold. If during the course of ECT seizure duration becomes less than 10 s, then modifications, based on the exact clinical situation will be made by the ECT psychiatrist in how ECT is provided in order to increase duration to above 10 s.

Per hospital protocol, if individuals experience cognitive impairment based on routine clinical evaluation with right unilateral or bilateral ECT during thrice a week sessions, ECT treatments may be performed twice a week (94). Cognitive impairment determined during routine clinical practice will be based on history and bedside clinical examination. If cognitive complications do not resolve at a twice a week frequency, right unilateral or bilateral ECT is transitioned to bifrontal ECT at the discretion of the clinical care team.

### High-density electroencephalography recordings during electroconvulsive therapy sessions

A high-density EEG recording system will be used to record signals before and after ECT charge delivery. A Net Amps 400 amplifier and Net Station software version 5.0 (Magstim, Eden Prairie, MN, USA) will be used to acquire EEG data sampled at 500 Hz. A network camera (Axis P3364LV, Axis Communications, Lund, Sweden) will be used to video record seizures during EEG recordings. Modified 64-channel GSN electrode caps (Magstim, Eden Prairie, MN, USA) will be used to ensure that ECT stimulation electrodes are appropriately placed for the procedure. Electrode sensors will be filled using electrode Elefix Ag/AgCl paste (Elefix, Nihon Kohden America, Inc., Irvine, CA, USA), to keep electrode impedances less than 100 kΩ per channel. Electrode sensors may be omitted if location will impact stimulation electrode placement (GSN E1, E4, and E54 during right unilateral ECT, E1, and E17 during bilateral ECT, E5, and E10 during bifrontal ECT).

Prior to induction of general anesthesia, separate 5-min recordings will be obtained for eyes-closed and eyes-open conditions. The EEG cap will be disconnected from the acquisition computer during electrical stimulation to protect the electrical equipment. After the ECT stimulus is delivered, the EEG cap will be reconnected. Our group has found that this method of disconnecting the cap generally leads to 1–2 s of lost data; this is comparable to the time of data lost using the standard 2-lead Thymatron IV System.

### Longitudinal recording of sleep electroencephalography using wireless wearable devices

Second-generation Dreem headbands will be used to acquire at-home unattended overnight sleep EEG recordings. The device has four frontal EEG electrodes [Fp1, Fp2 (ground), F7, and F8] and two occipital sensors (O1 and O2). The device has an infrared light source and sensor to measure heart rate. A three-axis set of accelerometers in the device allows monitoring of movement patterns that aid in sleep staging, arousal detection, and determination of respiratory effort. In the perioperative setting, this device provides adequate signals for detecting sleep spindles and sleep slow waves, with a single night sufficient for establishing baseline sleep macrostructure (27).

In addition to the Dreem headband, the option of wearing a wrist actigraphy device will be offered to allow evaluation of changes in circadian rhythms in a prespecified sub-study. ActTrust 2 wrist actigraphs (Condor Instruments, São Paulo, Brazil) will be utilized to collect longitudinal measures of rest and activity. These data will complement the longitudinal Dreem data and provide additional insight into sleep and circadian rhythm changes throughout ECT.

### Manual sleep scoring

Sleep EEG recordings will be imported and preprocessed using custom MATLAB (MathWorks, Natick, MA, USA) scripts and EEGLAB (95). Signals will be 0.5–50 Hz bandpass filtered and downsampled to 250 Hz. EEG signals and Dreem accelerometry respiratory effort waveforms will be exported in European Data Format (EDF). American Academy of Sleep Medicine (AASM) certified sleep technologists will then stage recordings using Philips Respironics Sleepware G3 Software and modified AASM scoring rules developed for use on single frontal EEG channel recordings (96, 97).

### Characterization of electroencephalography sleep microstructure

Custom-written MATLAB scripts will be utilized to compute sleep SWA and spindle density during 30-s epochs of N2 and N3. Sleep SWA will be calculated as an average of 0.5–4 Hz EEG power per minute during N3 sleep. As a secondary analysis, SWA during both N2 and N3 will be calculated. Dissipation of SWA over the overnight sleep will be evaluated through combining all NREM epochs, normalization of total sleep time by dividing total NREM length in quintiles, and comparison of SWA during the first and last quintiles (98). Spindle density will be quantified as the number of spindles per minute of N2 sleep. As a secondary analysis, spindle density for both N2 and N3 will be calculated. A semi-automated algorithm will be used to detect sleep spindles. This algorithm will be optimized based on partial manual scored spindles by registered polysomnographic technologists using AASM guidelines.

### Quantitative measures of electroconvulsive therapy-induced seizure markers

Recordings of ECT-induced seizures will be visually inspected and processed in Net Station Waveform Tools (Magstim, Eden Prairie, MN, USA). Data will be bandpass filtered at 1–100 Hz. Channels with excessive noise will be interpolated. Following previously established criteria (99), seizures will be staged across three phases by experienced epileptologists using Persyst software (Persyst, Solana Beach, CA, USA). Data from Phase 3, with rhythmic 2.5–3.5 Hz spike/polyspike-wave activity, will be utilized for CPC analysis using EEGLAB (95) in MATLAB. Custom MATLAB scripts will employ an automatic algorithm which will be utilized to detect large amplitude CPCs based on the difference between average suborbital facial electrode signals and Cz electrode (82). Cumulative duration of CPCs will be computed for each ECT session.

### Statistical analysis

Linear mixed-effects models will be constructed to account for missing data without the need for data imputation or deletion. Models will be generated in MATLAB. To characterize SWA over the course of ECT, linear mixed-effect models will be constructed using SWA as a dependent variable and treatment number as an independent variable. Similarly, to examine sleep spindle density changes, linear mixed-effects models will be generated that have sleep spindle density as a dependent variable and treatment number as an independent variable. Associations between CPC and sleep SWA or sleep spindles will be evaluated *via* respective linear mixed-effect models. For all aims, separate models will be constructed for responder and non-responder groups. QIDS-SR16 scores and ECT stimulation *parameters* will be entered as covariates for all analyses, as appropriate.

A total number of 25 participants will be targeted for recruitment in this study. Sample size calculations are based on the published data following conventions for statistical power analysis in the behavioral sciences (100) and our best guesses regarding effect size and SD. The main factor that we considered in estimating our required sample size was based on reported changes in SWA measure in major depression (98, 101, 102). We estimate a medium effect size and a partial η^2^ value of 0.1. Thus, we estimate sample sizes of 17 (power of 0.8, alpha of 0.05) based on a conservative assumption on the number of treatments (10 treatments) to account for missing a session or poor quality of EEG data. Participants may withdraw from the study at any time, and we will track reasons for patient attrition. Given that a previous study on a similar patient population cohort conducted at Barnes-Jewish Hospital and Washington University showed a 30% attrition rate (80), we expect to have at least 17 participants with interpretable data in the current study.

### Limitations

Technical limitations have impeded the study of longitudinal changes in sleep microstructure during the index course of ECT. While battery-operated wireless wearable devices allow continuous long-term EEG recordings in the inpatient and outpatient settings, these devices are equipped with dry electrodes that may be more prone to artifacts due to poor skin adherence. Thus, advanced filtering techniques are required to identify and suppress artifacts from EEG signals to fulfill the promise of this technology for large-scale sleep investigations in patients with mental health disorders. Another limitation of this study includes the potential existence of nonlinear relationships between dependent and independent variables in our analyses as different ECT stimulation montages may have posed different effects on sleep and seizure markers. These nonlinear relationships might not be explained by linear mixed-effect models. In such cases, generalized mixed-effect models will be utilized for data analyses. Another potential limitation in our study lies in the use of different classes and dosages of anesthetic agents and muscle relaxants during ECT sessions which may increase variance in data. Finally, while QIDS-SR16 has been reported to be a reliable and valid scale for assessing the severity of depression, it may contribute to sample variability compared to clinician-rated scales on depression such as HAMD or MADRS.

## Conclusion

The CET-REM study will probe longitudinal changes in sleep microstructure and seizure markers over the index course of ECT. Wireless wearable devices will be utilized for at-home unattended collection of sleep EEG to provide measures of sleep slow waves and sleep spindles. ECT-induced seizure markers will be assessed using high-density EEG recordings. Overall, the results from this study will elucidate underlying mechanisms and markers for responsiveness to ECT.

## Member of the CET-REM Study Group

Gaurang Gupte, L. Brian Hickman, Emma R. Huels, Allyson Quigley, and Maria Tello Borja.

## Ethics statement

The Washington University Human Research Protection Office (WU HRPO) reviewed and approved this study involving human participants. Informed consent will be obtained for all participants prior to the conduct of any study procedures. Written informed consent was obtained from the individual(s) for the publication of any identifiable images or data included in this article.

## Author contributions

All authors have made a significant contribution to the idea formation, study design, data acquirement, analysis and interpretation, and manuscript drafting and revision.

## Footnotes

1
https://clinicaltrials.gov/ct2/show/NCT04451135

## Abbreviations

AASM: American Academy of Sleep Medicine
BDNF: brain-derived neurotrophic factor
CPC: central-positive complexes
CET-REM: Correlating ECT Response to EEG Markers
DSR: delta sleep ratio
ECT: electroconvulsive therapy
EEG: electroencephalography
EDF: European Data Format
MDD: major depressive disorder
NREM: non-rapid eye movement
N1: NREM sleep stage 1
N2: NREM sleep stage 2
N3: NREM sleep stage 3
PSG: polysomnography
PSQI: Pittsburgh Sleep Quality Index
QIDS-SR16: Quick Inventory of Depressive Symptomatology-16 Self Report
SWA: slow wave activity
REM: rapid eye movement.
